# The assessment of efficacy of porcine reproductive respiratory syndrome virus inactivated vaccine based on the viral quantity and inactivation methods

**DOI:** 10.1186/1743-422X-8-323

**Published:** 2011-06-27

**Authors:** Hyunil Kim, Hye Kwon Kim, Jung Ho Jung, Yoo Jung Choi, Jiho Kim, Chang Gyu Um, Su Bin Hyun, Sungho Shin, Byeongchun Lee, Goo Jang, Bo Kyu Kang, Hyoung Joon Moon, Dae Sub Song

**Affiliations:** 1 Optifarm Solution Inc., 48 Songnam-ri, Seonggeo-eup, Cheonan, Korea; 2 College of Veterinary Medicine, Seoul National University, Seoul 151-742, Korea; 3 Green Cross Veterinary Products, Youngin 446-569, Korea; 4 Viral Infectious Disease Research Center, Korea Research Institute of Bioscience and Biotechnology, Daejon 305-806, Korea

## Abstract

**Background:**

There have been many efforts to develop efficient vaccines for the control of porcine reproductive and respiratory syndrome virus (PRRSV). Although inactivated PRRSV vaccines are preferred for their safety, they are weak at inducing humoral immune responses and controlling field PRRSV infection, especially when heterologous viruses are involved.

**Results:**

In all groups, the sample to positive (S/P) ratio of IDEXX ELISA and the virus neutralization (VN) titer remained negative until challenge. While viremia did not reduce in the vaccinated groups, the IDEXX-ELISA-specific immunoglobulin G increased more rapidly and to significantly greater levels 7 days after the challenge in all the vaccinated groups compared to the non-vaccinated groups (*p *< 0.05). VN titer was significantly different in the 10^6 ^PFU/mL PRRSV vaccine-inoculated and binary ethylenimine (BEI)-inactivated groups 22 days after challenge (*p *< 0.05). Consequently, the inactivated vaccines tested in this study provided weak memory responses with sequential challenge without any obvious active immune responses in the vaccinated pigs.

**Conclusions:**

The inactivated vaccine failed to show the humoral immunity, but it showed different immune response after the challenge compared to mock group. Although the 10^6 ^PFU/mL-vaccinated and BEI-inactivated groups showed significantly greater VN titers 22 days after challenge, all the groups were already negative for viremia.

## Background

Porcine reproductive and respiratory syndrome (PRRS) is an economically relevant emerging swine viral disease that was first recognized in North America in 1987 [1] and in Europe in 1990 [2]. The causative agent of this disease, PRRS virus (PRRSV), was first isolated in the Netherlands in 1990 [2] and was designated Lelystad virus (LV). Subsequently, the same agent causing PRRS was also identified in the United States [3]. PRRSV is an enveloped single-stranded RNA virus of the *Arteriviridae *family, a member of the order *Nidovirales *[4].

PRRSV can cause severe reproductive failure in sows that is characterized by late-term abortion, stillbirth, and the birth of weak piglets; it is also associated with porcine respiratory disease complex in combination with secondary infections [5,6]. In the US alone, the economic losses caused by PRRS amount to more than US $560 million annually, and it is the most significant infectious disease currently affecting the swine industry worldwide [7]. The application of vaccines against PRRSV began in 1993 in Europe and 1 year later in North America. Current PRRSV vaccines have 2 forms: modified live and inactivated virus mixed with adjuvant [8].

In breeding swine, post-vaccination viremia can be induced by this live vaccine, and boars can shed live vaccine virus in their semen [9]. A commercial modified live virus (MLV) vaccine is used to control PRRS in many countries, but PRRS outbreaks are quite common in swine farms despite routine vaccination. These findings justify studying the use of inactivated PRRSV vaccines in breeding pigs.

Because the protective immune response induced by attenuated vaccines is influenced by the genetic diversity of PRRSV, attenuated vaccines are not always effective against PRRSV that are genetically different from the vaccine virus strains [10]. For this reason, many researchers have tried to develop a killed virus vaccine that reflects the genetic diversity of PRRSV. Swenson et al. [9] show that use of a killed vaccine appears to reduce virus shedding in semen, but the difference in the number of days of shedding is not statistically significant compared to that of live vaccine. In contrast, Nielsen et al. [11] found that killed vaccine treatment has no effect on the level and duration of virus shedding in semen compared to live vaccine. Duran et al. [12] injected inactivated oily vaccine containing about 10^5.5 ^median tissue culture infectious doses (TCID_50_) per dose of a Spanish strain of PRRSV grown in porcine alveolar macrophages (PAMs) and subsequently challenged the cells with a live homologous strain. They showed that vaccinated animals devoid of antibodies, as determined by an immunoperoxidase monolayer assay (IPMA) at the time of challenge, were still protected from experimental PRRSV infection. On the other hand, despite the fact that Open reading frame 5 (ORF5) correlates well with the neutralizing antibody titer [13], the recombinant PRRSV ORF5 antigen vaccine did not produce serum-neutralizing antibodies and failed to show protection. Joo et al. [14] reported that sows of a PRRSV-positive herd with detectable serum neutralization (SN) antibody levels were not viremic after reexposure to PRRSV. Osorio et al. [15] showed that increased SN antibody titer is important, because the SN antibody response appears to be well correlated with resistance to infection. The SN antibody against PRRSV protects against viremia, virus replication in lungs [16], transplacental spreading of the virus, and reproductive failure [15].

Nilubol et al. [17] compared killed vaccine (KV)-inoculated infected pigs to infected pigs with and without KV inoculation. In this experiment, the SN titer was significantly higher in the KV-vaccinated groups after challenge than in the non-vaccinated groups. However, virus shedding was not affected. Misinzo et al. [18] reported a similar result in that the KV does not always induce neutralizing antibodies, but it enhances neutralizing antibodies upon viral challenge. Although KV induced faster antibody production after challenge, it failed to prevent the clinical signs associated with PRRSV infection, i.e., post-challenge viremia and transplacental infection of the piglets [19].

PRRSV-KVs induce poor immune responses in naïve pigs [17]. Without neutralizing antibody induction, KVs can result in a significant

improvement of sow reproductive performance and litter characteristics [20]. When animals were challenged with heterologous PRRSV, the vaccine failed to protect gilts [19]. The efficacy of inactivated PRRSV vaccine has been seriously questioned [21]. An effective KV program is thought to produce variable results, according to the vaccine and vaccination strategy. Misinzo et al. [18] observed differences in the efficacy of inactivated vaccines depending on the virus strain and the cells used to prepare the vaccines. In addition, some types of adjuvants can be used as effective vaccine adjuvants to enhance the humoral and cellular responses of piglets to PRRSV [22].

Despite frequent vaccine use, there is little existing information about the protective efficacy or potency of PRRSV vaccines evaluated through *in vivo *infectious challenge with wild-type PRRSV. This study was undertaken to compare vaccine efficacy according to the virus antigen quantity and inactivation reagent by analyzing the virus titer after challenge, ELISA antibody titer, and VN titer.

## Methods

### Virus production and plaque assay

PRRSV isolated from Jinwang farm in Chungcheongnam-do (virus isolation number: 08-296; Genbank accession number: HM130677) and propagated in MARC-145 cells was used for experimental vaccine preparation. After 96 h culture in 2-L roller bottles, the cell culture supernatants were centrifuged at 6,000 rpm for 20 min. Virus titers were determined by plaque assay using MARC-145 cells. For the assay, the cells were pre-seeded in a 6-well plate at a density of 3 × 10^5 ^cells/well for 12-18 h and subsequently infected with serial 10-fold dilutions of virus for 1 h at 37°C with frequent agitation. The cell monolayers were then overlaid with minimal essential medium containing 0.5% SeaKem LE agarose (FMC BioProducts, Rockland, Maine) and 5% fetal bovine serum, followed by 4-day incubation at 37°C in air containing 5% CO_2_. The resultant plaques were visualized by fixation with 7% formaldehyde followed by staining with crystal violet (1% [w/v] in 5% ethanol).

### Virus inactivation

#### 1. BEI

BEI was prepared as a 0.1 M stock solution by stirring 0.1 M 2-bromoethylamine hydrobromide (Sigma, USA) in 0.175 M NaOH at 37°C for 1 h, as described previously [23]. BEI was used shortly after its preparation. BEI stock solution was added to the virus suspension at 1% concentration to attain a final BEI concentration of 0.001 M. Virus suspensions were incubated at 37°C for 24 h. The remaining BEI was subsequently neutralized by the addition of 10% of the volume of BEI of 1 M sterile sodium thiosulfate (Sigma) solution for 2 h.

#### 2. Formalin

Formaldehyde solution (35%) (Duksan, Korea) was added to the viral suspensions to attain a final concentration of 0.3%. The formalin-treated viral suspensions were incubated overnight at 37°C as described previously {Habib, 2006 #856}.

#### 3. β-propiolactone

The pH of viral suspensions was adjusted to 7.3 using NaOH. β-propiolactone was then introduced to a final dilution of 1:2000 (v/v) and mixed on a magnetic stirrer for 24 h, as described previously [24]. Following inactivation, the viral suspensions were gradually heated to 37°C at pH 7.0 over a period of 24 h.

### Preparation of vaccine formulations

To confirm if the viruses were completely inactivated, 1 mL virus suspension was inoculated on MARC-145 cells in a 175-cm^2 ^tissue culture flask with 50 mL of Dulbecco's modified Eagle's medium (DMEM). After the cells were cultured for 5 days at 37°C, the supernatants were replaced with fresh culture media and incubated for another 5 days. Non-inactivated PRRSV was inoculated on MARC-145 cells as positive control. Cells were analyzed for cytopathic effects (CPE), and the supernatants were tested by polymerase chain reaction (PCR) to confirm the presence of the virus. After confirmation of viral inactivation, each viral antigen were mixed with 10% (v/v) aluminum hydroxide adjuvant (Rehydragel, SEPPIC, France) on a magnetic stirrer (at 180 rpm) for overnight.

### PRRS ELISA

Serum samples were collected from pigs, and aliquots were prepared and stored at -20°C until ELISA for PRRSV antibodies was performed. ELISA was performed using a commercial kit (HerdChek^® ^PRRS 2XR, IDEXX), according to the manufacturer's instructions. All reagents required for the assay were provided with the kit, and the assay was conducted at room temperature. The optical density of each well was measured at 650 nm using the Bio-Rad 680 microplate reader. The presence or absence of PRRSV antibody was determined by calculating the sample to positive (S/P) ratio. Samples were considered to be positive for PRRSV antibody if the S/P ratio was more than 0.4.

### PRRSV VN titer assay

VN titers were determined by SN test on MARC-145 cells. A 2-fold diluted serum sample was prepared, and an equal volume of virus solution with a titer of 100 TCID_50_/mL was added to each dilution and incubated for 1 h at 37°C. The serum-virus mixture was transferred to a 96-well plate containing a MARC-145 cell monolayer. The CPEs on the cells were analyzed for 7 days after inoculation. The VN antibody titer was defined as the reciprocal of the highest dilution that inhibited CPE in 50% of the inoculated wells.

### Quantitation of PRRSV with real-time PCR

Real-time quantitative PCR was performed using Bio-Rad iQ5 Real Time PCR Detection System. The 20-μL PCR mixture comprised 10 μL of the commercially available mastermix (iQ SYBR Green Supermix; Bio-Rad), 1 μL of the cDNA extract from the serum of each pig, 8.5 μL of RNAase-free water, and 0.25 μL of both forward and reverse primers. In addition, each reaction included PRRSV standards with progressive dilutions of 1:10, which generated the standard curve for the reaction. The following protocol was used: 5 min at 94°C for incubation and Taq activation, followed by 40 cycles of 30 s each at 94°C for denaturation, 30 s at 55°C for annealing, and 45°C for extension. PRRSV content in each sample was estimated by converting the value for the cycle threshold (Ct), which was determined with the Bio-Rad iQ5 qPCR software, to virus titer (copies/mL) by using the coefficient of correlation from the standard curve.

### Experimental design of animal studies

All animals were serologically tested against PRRSV just before the experiment to confirm naïve herd status. All animal experiments complied with the current laws of South Korea. Animal care and treatment were conducted in accordance with the guidelines established by the Institutional Animal Care and Use Committee of Optifarm Solution. Twenty-eight SPF hairless white Yucatan miniature pigs were used in this experiment. All pigs were kept in a HEPA-filtered barrier facility. The pigs had free access to sterilized water and unmedicated sterilized feed.

Twenty-eight SPF miniature pigs were randomly assigned to 7 treatment groups including a negative control group. The mock-vaccinated control group received 2 mL aluminum hydroxide adjuvant. The 10^4^, 10^5^, and 10^6 ^groups were vaccinated with 10^4^, 10^5^, and 10^6 ^PFU/mL inactivated PRRSV (by the BEI method), respectively, with 10% (v/v) aluminum hydroxide adjuvant (Rehydragel, SEPPIC, France). The BEI-, formalin-, and β-propiolactone-inactivated groups were vaccinated with PRRSV inactivated by the abovementioned compounds. The virus titer in the vaccine was 10^6 ^PFU/mL, which was mixed with 10% (v/v) aluminum hydroxide adjuvant.

All the 7 groups were inoculated with vaccine or mock-vaccine 3 times 0, 21, and 42 days post inoculation (DPI). At day 70 after initial vaccination, all the animals (the mock, 10^4^, 10^5^, and 10^6 ^groups) were challenged intranasally with 10^3.4 ^TCID_50 _field-isolated PRRSV (strain 09-1240 KH, ORF5; Genbank accession number: GQ375443). Blood was taken on 0, 3, 6, 9, 14, 21, 28, 35, 42, 49, 56, and 70 days after vaccination and on 0, 3, 4, 7, 10, 15, and 22 days after the challenge. Serum samples were collected and stored at -70°C before testing with ELISA, VN, and Q-PCR.

### Statistical analysis

ELISA S/P ratio, V/N test results, and viral load were analyzed by Student's *t*-test. A *p *value less than 0.05 was considered statistically significant.

## Results

### Measurement of PRRSV-specific antibody levels by IDEXX ELISA after vaccination according to the viral titer

**Figure 1:**
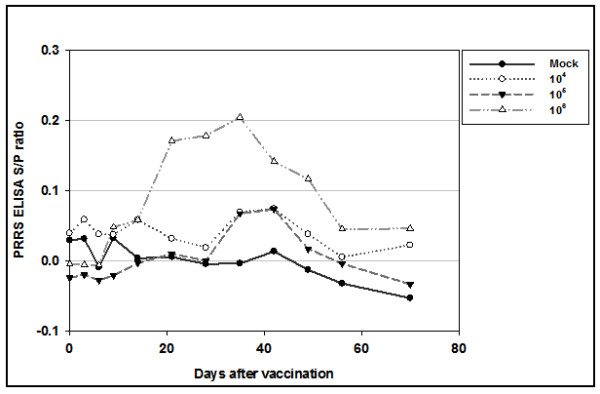
**PRRSV-specific IDEXX ELISA S/P ratio with 3 shots of BEI-inactivated PRRSV experimental inactivated vaccine, according to viral titers**. Vaccines were inoculated 3 times (days 0, 14, and 28).

For the experimental efficacy test according to the viral titer, PRRSV suspensions of titers of 10^4^, 10^5^, or 10^6 ^PFU/mL were inactivated with binary ethylenimine (BEI) and inoculated into pigs. From 21 to 56 days after the first vaccination, the 10^6 ^PFU/mL PRRSV vaccine-inoculated group showed a significantly higher sample to positive (S/P) ratio (*p *< 0.01) than the control group (Figure 1). However, all groups were serologically negative as determined by IDEXX ELISA until 70 days of the first vaccination. The positive and negative cut-offs of the IDEXX ELISA are at 0.4 S/P ratio. The S/P ratio of the 10^6 ^PFU/mL PRRSV vaccine-inoculated group had the highest S/P ratio at 28 day post-infection (DPI) 28, 14 days after the second injection. This result indicated that 10^6 ^PFU/mL killed PRRSV antigen could not induce enough antibody determined by ELISA.

### Variation of PRRSV ELISA S/P ratio after challenge according to the virus titers

**Figure 2:**
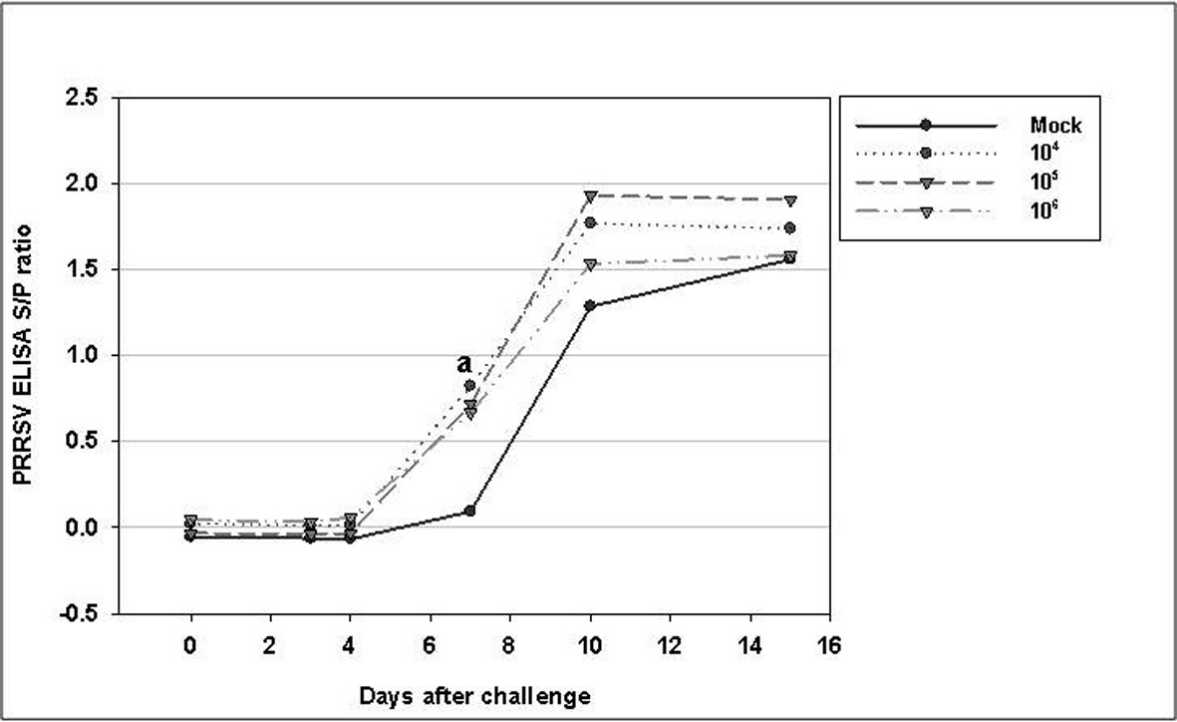
**PRRSV-specific antibody titer (ELISA S/P ratio) after challenge**. The 4 groups were challenged at day 70 post-primary vaccination. The values shown correspond to the average S/P ratio at each time point. ^a ^Mock group was significantly different from other groups (*p *< 0.05).

PRRSV-specific antibodies were found to be absent by ELISA in all the experimental groups before challenge (Figure 1). Until day 4 after challenge, all the groups were serologically negative as determined by IDEXX ELISA (Figure 2). At day 7 after the challenge, all the vaccinated groups became serologically positive. The average S/P ratios of the 10^4^, 10^5^, and 10^6 ^PFU/mL PRRSV-vaccinated groups at 7 days were 0.82, 0.72, and 0.67, respectively. The average S/P ratios of the 10^4 ^and 10^5 ^PFU/mL vaccine-inoculated groups were not significantly different from the 10^6 ^PFU/mL vaccine-inoculated groups (p > 0.05). However, all vaccine groups showed significantly different results from the control group only on the seventh day after challenge (*p *< 0.01). This result indicates that, all vaccinated groups showed faster antibody production than the non-vaccinated group.

### Viral load after challenge with killed vaccine according to viral titer

**Figure 3:**
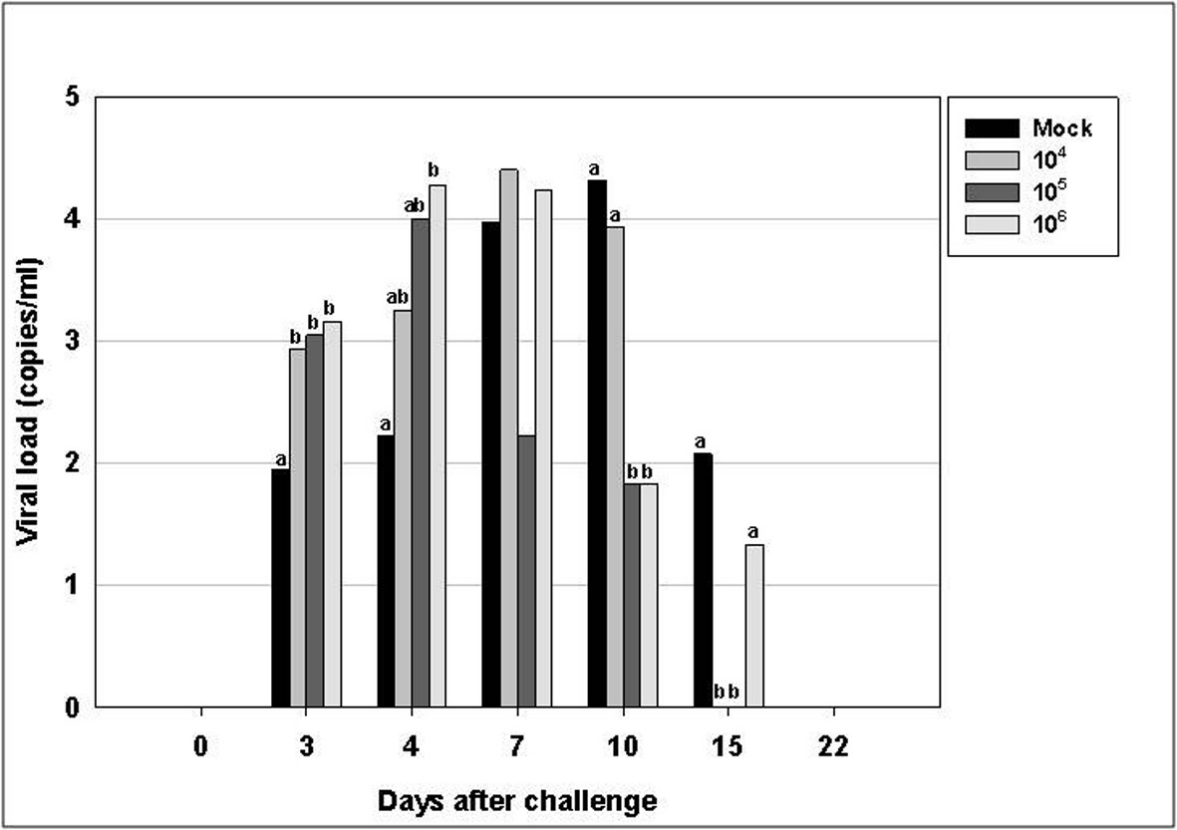
**PRRSV load in serum samples collected after challenge from both vaccinated and control (unvaccinated) groups**. Results were obtained by quantitative PCR and are expressed as copies/mL serum. The 4 groups were challenged at day 70 post-primary vaccination. Bars represent the average viral load at each time point. Values with superscripts in each experimental day are significantly different (*p *< 0.05).

The mean post-challenge serum PRRSV level is shown in Figure 3. The sensitivity threshold of this assay is 10 RNA copies/mL measured by quantitative PCR.

Because day-0 blood was sampled just before inoculation, the virus was not detected. By day 3 after challenge, all the vaccinated groups showed significantly different results from the control group (*p *< 0.05). The viral load of the control group was lower than those of the vaccinated groups. On day 4, the viral load of the 10^6 ^PFU PRRSV-vaccinated group was significantly higher than that of the control group; no significant differences were observed compared to that of the other groups (p < 0.05). At day 10, the viral loads of the 10^5 ^and 10^6 ^PFU/mL PRRSV-vaccinated groups were significantly reduced compared with those of the control and 10^4 ^groups (*p *< 0.05). There was no difference in viral load between the control and 10^4^, 10^5^, and 10^6 ^PFU/mL PRRSV-vaccinated groups. By day 15, viruses were detected in the control and 10^6 ^PFU/mL PRRSV-vaccinated groups, but there was only a significant difference between the control and 10^4 ^PFU/mL PRRSV-vaccinated groups (*p *< 0.05).

### PRRSV-specific neutralization titer according to BEI-inactivated viral titers from the samples on day 22 after challenge

**Figure 4:**
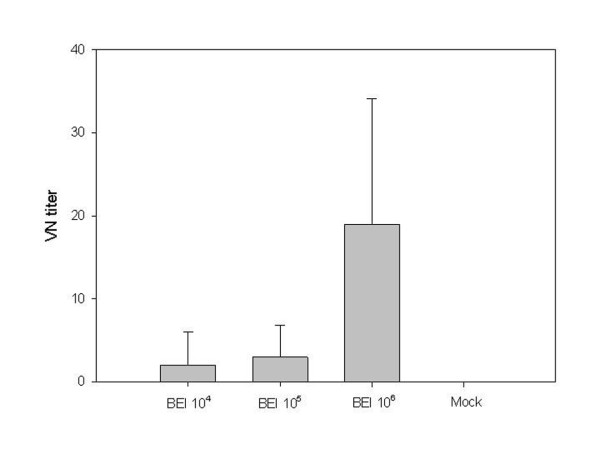
**Virus-specific neutralization titer according to BEI-inactivated viral titers from the samples collected 22 days after challenge**. The virus neutralization test titer of the 10^6 ^viral antigen-inoculated group was significantly greater than those of the 10^4 ^and 10^5 ^PFU/mL virus-inoculated groups (*p *< 0.05).

On the last day after challenge, serum samples were analyzed to determine the difference in the VN titer obtained with different values of the inoculated viral titer. Before challenge, all experimental groups were measured by a VN test and all groups were negative. Twenty-two days after challenge, the 10^6 ^viral antigen-inoculated group exhibited a significantly higher VN titer compared to the 10^4 ^and 10^5 ^PFU/mL virus-inoculated groups (p < 0.01). In this present study, killed vaccine failed to induce antibody determined by ELISA. But BEI-inactivated vaccines showed higher VN titer according to viral titer. This result indicated that 10^6 ^PFU/mL viral titer of vaccine can induce higher VN titer (Figure 4).

### PRRSV-specific ELISA antibody titer with experimental vaccines according to inactivation methods

**Figure 5:**
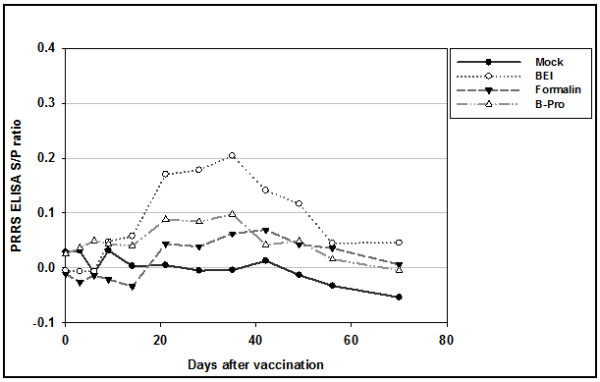
**PRRSV-specific IDEXX ELISA S/P ratio with 3 shots of experimentally inactivated PRRSV vaccine, according to inactivation method (BEI, formalin, and β-propiolactone)**. Vaccines were injected 3 times (day 0, 14, and 28).

In order to test the efficacy of different virus inactivation methods, PRRSV suspensions were inactivated using BEI, formalin, or β-propiolactone. Until 70 days after the first vaccination, all experimental groups showed serological negativity (Figure 5), as determined by the IDEXX ELISA kit. The positive and negative cut-offs of the IDEXX ELISA were at an S/P ratio of 0.4. The BEI-inactivated group showed significantly different S/P ratios compared to the control group from 21 to 56 days after the first vaccine inoculation (*p *< 0.01). The highest S/P ratio was observed in the BEI group 14 days after the second vaccination and 28 days after the primary vaccination (0.222 (0.1)). In the formalin group, from 28 to 42 days, only 1 pig (H08-010) had a serologically positive S/P ratio above 0.4, and its highest value in that period was 0.68.

### Variation in PRRS ELISA S/P ratios after challenge with vaccines prepared by different virus inactivation methods

**Figure 6:**
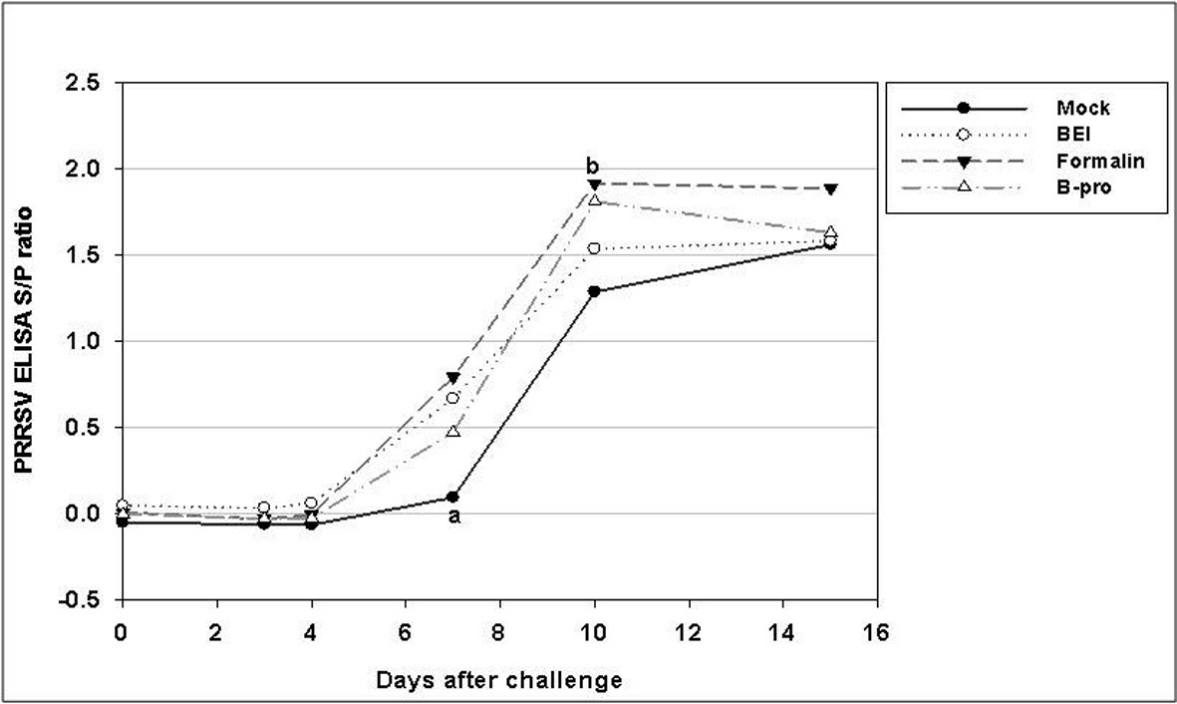
**PRRSV-specific antibody titer (ELISA S/P ratio) after challenge**. The 4 groups were challenged 70 days post-primary vaccination. Values represent the average S/P ratio at each time point. ^a ^Formalin group is significantly different from BEI and mock groups (*p *< 0.05).

The PRRSV-specific antibodies were found to be absent by ELISA in all the experimental groups before challenge (Figure 5). Until day 4 after challenge, all the groups remained serologically negative, as determined by IDEXX ELISA (Figure 6). At day 7 after challenge, the average S/P ratio of all vaccinated groups became serologically positive, except for 1 and 2 pigs in the formalin-and β-propiolactone-inactivated groups, respectively.

On the seventh day after challenge, all vaccinated groups showed significantly different S/P ratios compared to the control group (*p *< 0.01). The S/P ratio of the formalin-inactivated group on the tenth day was significantly different from that of the control group (*p *< 0.005). There were no significant differences 15 days after challenge.

### Viral load of the KV-vaccinated group studied according to the different method of virus inactivation

**Figure 7:**
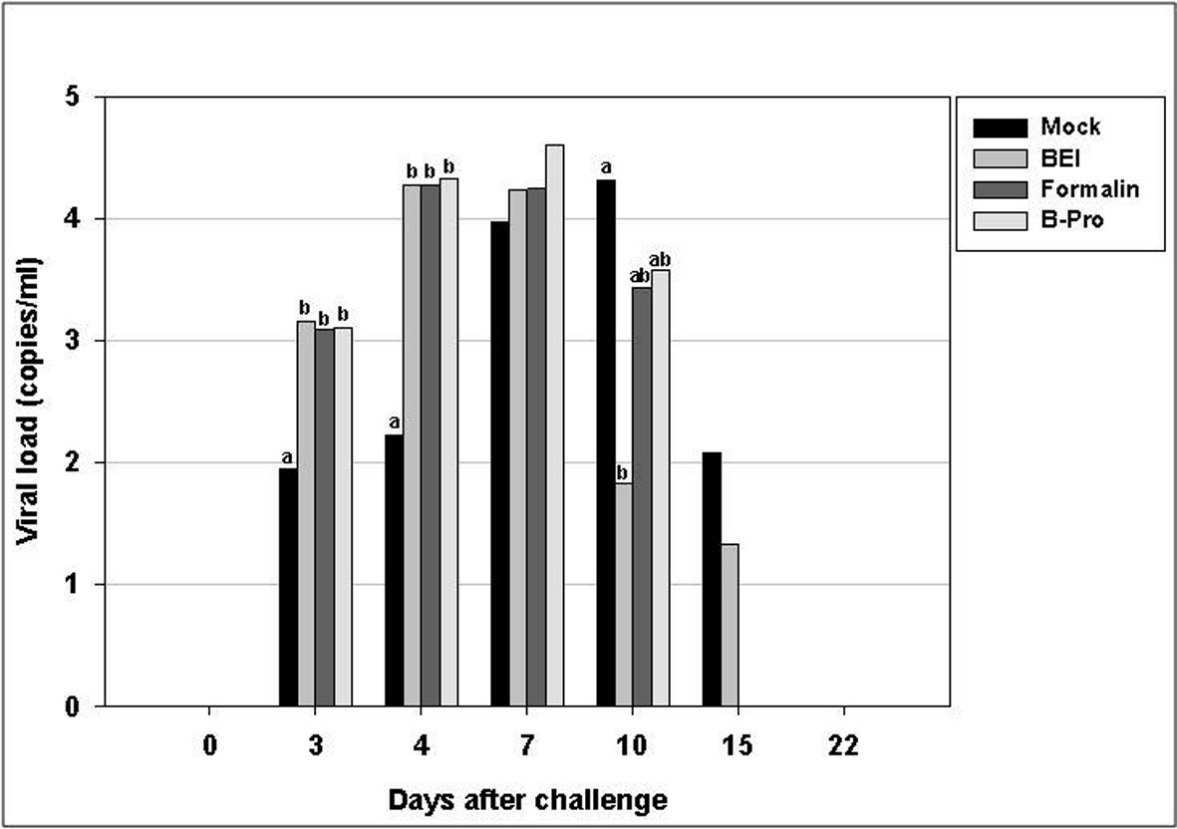
**PRRSV load in serum samples after challenge**. The results were obtained by quantitative PCR and are expressed as copies/mL of serum. The 4 groups were challenged on day 70 after primary vaccination. Bars represent the average viral load at each time point. Values with superscripts in each experimental day are significantly different (*p *< 0.05).

The mean PRRSV load in the serum samples collected after the challenge is shown in Figure 7. The threshold of sensitivity of this assay was 10 RNA copies/mL measured by quantitative PCR.

The virus was not detected, because the blood samples were taken just before the challenge. From days 3-4, all the vaccinated groups, except the β-propiolactone-inactivated group at day 3, had significantly higher viral loads than the control group (*p *< 0.05). Ten days after the challenge, the BEI-inactivated group had a lower viral load than the control group (*p *< 0.05). Twenty-two days after the challenge, only the control and BEI-inactivated groups had virus-positive blood.

### PRRSV-specific neutralization titer according to virus inactivation method from the samples collected 22 days after challenge

**Figure 8:**
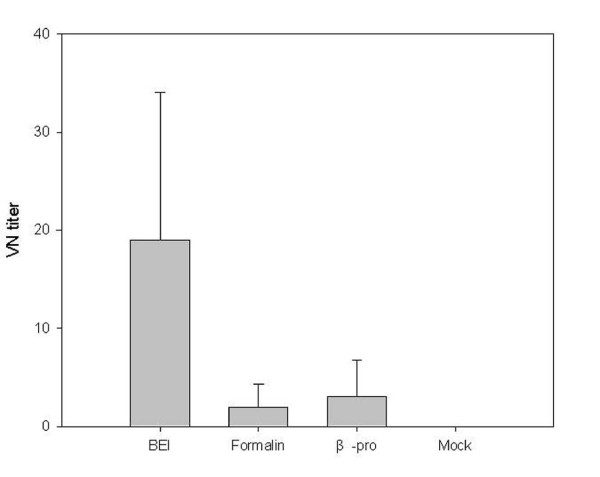
**Virus-specific neutralization titer according to virus inactivation method using the samples 22 days after challenge**. The virus neutralization test titer of the BEI-inactivated group was significantly greater than the formalin- and β-propiolactone-inactivated groups (*p *< 0.05).

On the last day after challenge, the serum samples were analyzed to determine differences in VN titer, according to inactivation method used to prepare vaccines (Figure 8). Before challenge, all experimental groups were measured by VN test, and all groups were negative. Twenty-two days after the challenge, the BEI-inactivated group had a significantly higher titer compared to the formalin- and β-propiolactone-inactivated groups (p < 0.01).

## Discussion

The total annual economic impact of PRRS on the US swine producers is estimated at $66.75 million in breeding herds and $493.57 million in growing pig populations [7]; the situation in South Korea is the same. Based on a field survey in 2001, 230 of 256 pig farms in Korea tested positive (89.8%) for PRRSV antibodies [25], indicating that PRRSV has spread throughout the country. Thus, for preventive purposes, MLVs costing as much as 2.5 billion KRW per year are used in South Korea. Despite the use of live vaccines, the prevalence of PRRSV has not decreased dramatically. This is because attenuated vaccines are not always effective against PRRSVs that are genetically different from the vaccine virus strain [10]. The PRRSV ORF5 sequence surveillance data since 2007 identified newly emerging PRRSVs (MN184-like) that have 84.9-87.2% nucleotide similarities compared to VR-2332, based on ORF5 sequence [26]. Recently, attenuated and inactivated vaccines have been introduced to control PRRSV in the field. Considering their safety and flexibility towards emerging virus strains, KVs are preferred over attenuated vaccines. However, in spite of these benefits, KVs have failed to evoke any measurable protective immunity [8] or protect against clinical signs such as post-challenge viremia and transplacental infection [19].

The first purpose of this study was to investigate the effects of experimentally KVs of varying virus titers on humoral immunity. In this experiment, PFU/mL was used as the viral particle-measuring method to obtain a more accurate count. The killed PRRSV vaccine was inoculated 3 times into SPF miniature pigs at 3-week intervals. Although all pigs were seronegative according to the criteria provided by the IDEXX corporation (S/P ratio < 0.4), the KV group inoculated with 10^6 ^PFU/mL had significantly higher S/P ratios than the control group (p < 0.05). However, no VN titers were detected in the entire experimental period before challenge (data not shown).

Many researchers have tried to establish the effects of KVs on humoral immunity. Misinzo et al. [19] investigated whether neutralizing antibodies can be induced in pigs upon vaccination with an inactivated vaccine. The antibody measuring protocols, which differ from researcher to researcher, include SN titer [17], IPMA [18], serum immunoglobulin G [22], and ELISA antibody titer [8,19]. In these studies, the effects of KVs on humoral immunity were undetectable or were detected at a very low level by using commercial ELISA systems. There might be a difference between the major antigenic epitopes of killed PRRSV vaccines and the ELISA system. However, Scortti et al. [19] reported that the first seroconversion resulted with the inoculation of Suvaxyn (Fort Dodge) KV. By day 21, all vaccinated gilts had seroconverted with a geometric mean titer (GMT) of 7.43 (0.44), and a peak of anti-PRRSV antibodies measured by ELISA was observed on day 70 with a GMT of 8.22 (0.54) [27]. These varying results allow the assumption that there are many obstacles to establishing effective KV combinations and well-matched diagnostic methods.

In the challenge experiment of the present study, all vaccinated groups showed faster antibody production than the control group. Scortti et al. [19] reported that vaccinated groups produced detectable antibodies 5 days after challenge and 9 days before the unvaccinated group. In the present study, PRRSV-specific antibodies were detected at 7 days, that is, 3 days before the control group. This may imply that the killed vaccine can induce enough immune memory for PRRSV, but not enough to induce active humoral immunity. These findings are very similar to those of Zuckermann et al. [8]; only the challenge time is different (28 days post-primary vaccination versus 70 days in the present study). The more rapid development of a post-challenge serological response may confer some degree of protective immunity in pigs [19]. The key point of the present study is that all the vaccinated groups, which had different viral antigen concentrations ranging from 10^4 ^PFU/mL to 10^6 ^PFU/mL, induced the same pattern of more rapid humoral response. Only with this data, the 10^4 ^level of viral antigen inoculation appeared to be sufficient for inducing partial immunity. However, the VN titer measured 22 days after challenge showed that only the 10^6 ^PFU/mL virus-inoculated group had significantly greater neutralizing antibody titer than the other groups (p < 0.05). Thus, if this viral neutralization response is related to protective immunity, it is also expected to affect the post-challenge serum viral load. In another study, KV (10^5.5 ^TCID_50_)-inoculated animals showed a shorter period of viremia, indicating that it is possible that the virus concentration in the blood of vaccinated animals is lower than that in the non-vaccinated animals [12].

Misinzo et al. [18] reported that the incomplete protection of KV vaccines against PRRSV might be caused by over-inactivation, resulting in the destruction of neutralizing viral epitopes. Virus inactivation procedures can affect the conservation of inactivated viral epitopes that are important for the induction of protective immunity [28]. In the present study, the ELISA S/P ratios of all vaccinated groups were significantly greater than those of the control group (p < 0.01). Because BEI has an effect at the genomic level--specifically for nucleic acids--preserving viral epitopes [28], the BEI-inactivated group might exhibit a higher S/P ratio than the other groups. However, BEI had very little adverse effects on the epitopes, whereas BPL significantly altered and formalin partially altered the conformation of most epitopes [29]. All the vaccinated groups showed significant rapid elevation of antibody levels at day 7 (Figure 5), and the formalin-inactivated group showed significance at day 10 compared to the control group (*p *< 0.01). However, in the VN test, BEI resulted in a significantly higher VN titer than other inactivation groups. At least in this experiment, the inactivation method is considered a key factor affecting the VN titer of inactivated PRRSV vaccines.

Unexpectedly, in the challenge experiment after vaccination according to the viral titer, the vaccinated groups showed higher viral loads than the control group until day 4. This phenomenon was repeated in the challenge experiment after vaccination according to inactivation method. Scortti et al. [19] found higher viral loads than those in a mock-vaccinated group after 3 and 5 days. In contrast, [30] found lower viral loads in the vaccinated group than in the mock-vaccinated group. The reason why vaccinated groups exhibit significantly higher viral loads in blood is unclear. However, it is clear that while vaccination with inactivated viruses only has a small effect on viremia levels, it reduces the duration of viremia as reported by other researchers [18].

## Conclusions

In conclusion, killed PRRSV vaccines with different concentrations of virus titer or based on different inactivation protocols did not significantly differ in their ability to induce humoral immunity in pigs even after 3 inoculations. However, all the vaccinated groups reached seropositive status in IDEXX ELISA much before the non-vaccinated group after challenge, which suggests that the KV merely potentiates memory response. Although the 10^6 ^PFU/mL-vaccinated and BEI-inactivated groups showed significantly greater VN titers 22 days after challenge, all the groups were already negative for viremia. Thus, the BEI-inactivated vaccine with a greater virus titer should be considered for testing to evaluate the efficacy of killed PRRSV vaccines.

## Competing interests

The authors declare that they have no competing interests.

## Authors' contributions

The formulation of the study was by JJ, YC, and HK. Animal experiments were by JK, CU and SH. Results were analyzed by SS. Revision by BL and GJ. Scripting was by BK, HM, and DS. All authors read and approved the final manuscript.
